# Familial pancreatic cancer: a case study and review of the psychosocial effects of diagnoses on families

**DOI:** 10.1186/s13053-023-00261-5

**Published:** 2023-09-08

**Authors:** Tracy Lowe, Jane DeLuca, Ludovico Abenavoli, Luigi Boccuto

**Affiliations:** 1 School of Nursing, Clemson University, Clemson, SC 29634 USA; 2 Clemson, USA; 3 Gastroenterology, Department of Health Sciences, University Magna Graecia, 88100 Catanzaro, Italy

**Keywords:** Familial pancreatic cancer, Genetics, Screening, Psychosocial impact

## Abstract

**Background:**

Familial pancreatic cancer touches families through a genetic susceptibility to developing this neoplasia. Genetic susceptibility is assessed via family history, genetic testing, or both. Individuals with two or more first-degree relatives or three or more relatives of any degree diagnosed with pancreatic cancer are considered at elevated risk. Following a diagnosis of familial pancreatic cancer, patients and families face uncertainty and anxiety about the future. Psychosocial effects of a pancreatic cancer diagnosis on families include fear, concerns about personal health, and how lifestyle may impact the risk of developing pancreatic cancer.

**Case presentation:**

A 66-year-old male was diagnosed with pancreatic ductal adenocarcinoma stage IIB, T3, N1, M0. A genetic referral was made due to a history of multiple cases of pancreatic cancer within the patient’s family. Genetic testing revealed the patient had a pathogenic variant in the *ATM* gene that is associated with an increased risk for pancreatic cancer development. The patient’s one adult child was offered testing due to the autosomal dominant pattern of inheritance for this variant. The adult child was found to have the same pathogenic variant. She expressed fear for her future and her child’s future health and longevity. Discussing a case study allows us to capture the multi-faceted relationship between the disease, the affected individuals, and their families. Examining the psychosocial stresses and concerns when there is a pancreatic cancer diagnosis in the family is essential to provide holistic care to patients and families.

**Conclusions:**

The psychosocial effects of FPC may be overwhelming for patients and families. Healthcare providers can offer education, support, and referrals to appropriate services to help families cope through stages of evaluation, diagnosis, and treatment of FPC.

Pancreatic cancer (PC) is the third leading cause of cancer-related deaths in the United States, with a 95% mortality rate within five years of diagnosis [1]. The yearly prevalence rate of PC is increasing, and it is estimated that it will be the second deadliest cancer by 2030 [1]. PC’s low survival rate is related to its ability to proliferate without signs and symptoms. Once diagnosed, the neoplasm is often at an advanced stage with a poor prognosis [2]. Identifying individuals at risk for PC is essential in order to implement routine screening, which can lead to early disease identification and intervention.

Approximately 10% of PC diagnoses are hereditary, meaning individuals have a genetic variant that increases their risk of developing pancreatic cancer. Familial pancreatic cancer (FPC) is diagnosed in individuals with two or more first-degree relatives or three or more relatives of any degree with pancreatic cancer [3]. Individuals with these pathological variants have a 50% chance of passing that pathogenic variant to their children. Persons with certain conditions, such as Peutz-Jeghers syndrome, hereditary pancreatitis, hereditary breast and ovarian cancer syndrome, have a higher risk associated with pancreatic cancer development [4]. Non-genetic risk factors that are associated with the development of pancreatic cancer include smoking, diabetes, obesity, chronic pancreatitis, pancreatic cysts, and additional forms of neoplasms [5].

One priority for improving PC outcomes is to develop ways to detect it earlier. Genetic testing can identify individuals with pathogenic variants associated with the development of pancreatic cancer. One study found the public was generally positive toward genetic testing and counseling [6]. However, there was less overall awareness of genetic testing in determining cancer risk among different ethnic groups including persons of African American, Hispanic, and Asian descent [6]. Early detection may improve survivability. Identifying at-risk individuals through genetic testing is imperative, as many cases of FPC are related to specific genetic variants, which can be passed genetically through families [7]. Genes associated with FPC development include pathogenic variants in *ATM*, *BRCA1*, *BRCA2*, *PALB2*, and *CDKN2A* genes. Other genes, such as *STK11*, *MLH1*, and *MSH2,* are also related to FPC susceptibility [8].

A PC diagnosis within a family has important ramifications for at-risk family members’ health beliefs and behaviors. Early identification of variant gene carriers may empower individuals to make informed decisions regarding health behaviors and surveillance strategies. Following the diagnosis of a loved one, family members may experience detrimental psychosocial effects. Understanding the implications of FPC and how it psychologically affects genetically at-risk individuals can aid in providing comprehensive and holistic patient care to families. This paper aims to present an FPC case study and examine the psychosocial stresses and concerns when there is a PC diagnosis in the family.

## Case report

### Chief complaint and history of present illness

A 66-year-old man presented to the gastroenterologist after sudden onset jaundice, dark urine, and light-colored stools, which began several days before the visit. The patient lost approximately twenty pounds over the previous weeks without dieting. He complained of feeling full after eating only a small amount of food. The patient denied any nausea, vomiting, or abdominal pain.

### Medical history

The patient had no known allergies. He had chronic obstructive pulmonary disease (COPD), hypertension, and gastroesophageal reflux disease (GERD), all well-controlled with medication. Current medications include albuterol inhalers for bronchospasms, amlodipine for hypertension, and omeprazole for GERD. The patient previously had an appendectomy at age six and a cystoscopy for kidney stone treatment at age 27. The patient had no previous history of broken bones or physical trauma.

### Social history

The patient has been married for 48 years. He currently uses chewing tobacco daily. He had a history of smoking 1.5 packs of cigarettes per day for 40 years but stopped smoking three years ago. He had a history of daily alcohol use, primarily beer, but stopped drinking alcohol two years ago.

### Family history

The patient’s mother is living and has a history of hypertension and GERD. His father died at the age of fifty-six from hepatocellular carcinoma. The patient’s brother, who lives locally, was diagnosed with pancreatic cancer three weeks before the patient’s evaluation. His maternal and paternal grandparents were deceased. His maternal grandfather was deceased at age 80 from prostate cancer. His maternal grandmother was deceased at age 73 from complications related to type 2 diabetes. Information about the causes of death for his paternal grandparents was unavailable.

### Physical examination

The patient was alert and oriented. Physical examination revealed visible jaundice of the sclera and skin, but his skin was otherwise clear, with no lesions, bruising, or bleeding. The patient’s head, ears, nose, and throat appeared normal. His lungs were clear to auscultation, and his heart had a regular rate and rhythm. His abdomen was soft, non-distended with no ascites, and non-tender to palpation. The patient’s extremities showed no signs of edema. He had symmetrical muscle strength and a normal neurological exam.

### Medical work-up

Blood tests were ordered based on the patient’s history of present illness and physical examination. A complete metabolic panel, prothrombin time, and alpha-1-antitrypsin were completed and showed abnormal findings (Table 1). A computed tomography (CT) scan of the abdomen and pelvis with and without contrast was ordered. The CT revealed a mass of 4-cm diameter in the pancreatic head obstructing the common bile duct. There were no hepatic masses or metastases found. The patient underwent endoscopic retrograde cholangiopancreatography (ERCP) for biopsy and staging.

**Table 1:** Laboratory findings in the reported patient

**Laboratory Test**	**Findings**	**Normal Ranges**
**Complete Blood Count with Differential**	*Neutrophil 84.0%*	40.0–60.0%
	*Lymphocyte 12%*	20.0–40.0%
**Complete Metabolic Panel**	*Glucose 260 mg/dL*	70–100 mg/dL
	*Sodium 134 mEq/L*	135–145 mEq/L
	*Chloride 92 mEq/L*	96–106 mEq/L
	*Total protein 5.3 g/dL*	6.0–8.3 g/dL
	*AST (SGOT) 168 IU/L*	8–33 IU/L
	*ALT (SGPT) 268 IU/L*	3–36 IU/L
	*Alkaline Phosphatase 1182 IU/L*	20–130 IU/L
	*Total Bilirubin 14.3 mg/dL*	0.1–1.2 mg/dL
**Prothrombin time**	*25.7 s*	11–13.5 s
**Alpha-1-antitrypsin, quantitative**	*211 mg/dL*	75–150 mg/dL

### Diagnosis

The patient was diagnosed with pancreatic ductal adenocarcinoma stage IIB, T3, N1, M0 (Table 2). Additionally, he was diagnosed with new-onset type II diabetes mellitus.

**Table 2:** Pancreatic ductal adenocarcinoma staging

**IIB**	Spread outside of the pancreas to local peripancreatic tissues
**T3**	2–4 cm sized tumor
**N1**	Spread to regional lymph nodes
**M0**	No distant metastasis

### Treatment plan

The patient was referred to a medical oncologist for chemotherapy. He was referred to a genetic clinic to evaluate genetic risks and to undergo genetic testing.

### Genetic referral

**Fig. 1:**
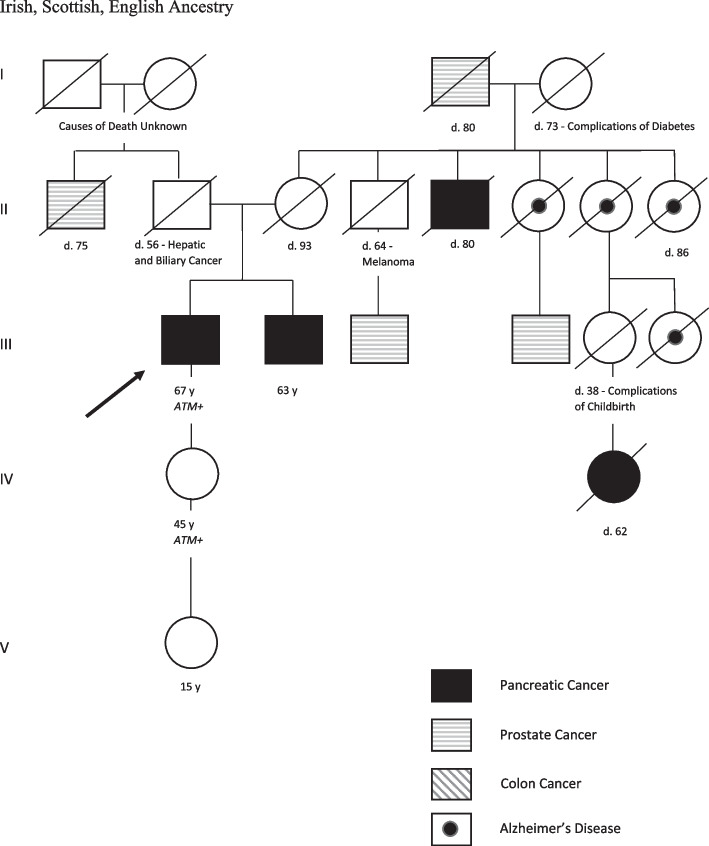
Family history drawing

The patient and family met with a genetic counselor who completed a family pedigree (Fig. 1). The pedigree revealed a history of PC on the patient’s maternal side. In addition to his brother, the patient had a maternal uncle who died from PC at age 80 and a second cousin who died from PC at age 62. His maternal grandfather died of prostate cancer at age 80. His uncle and second cousin lived less than a year after diagnosis. Additionally, two first cousins on his maternal side were diagnosed with prostate cancer.

### Outcome of genetic referral

Based on his diagnosis and family history, the genetic counselor offered the patient genetic testing. The patient underwent testing for genes associated with FPC. The patient tested positive for a pathogenic change in the *ATM* gene. This gene change is inherited in an autosomal dominant pattern, meaning the patient’s children have a 50% chance of having the pathogenic variant. Genetic testing was offered to the patient’s only child.

The patient’s adult child underwent testing and was found to have the same pathogenic variant as her parent. She expressed fear of one day developing PC and was concerned for her child’s future health and longevity.

### Case summary

Gastroenterology services evaluated the patient for rapid onset of jaundice and weight loss. He was found to have abnormal laboratory values suggestive of a cancer process, with identification and biopsy of a mass. The family history was positive for several forms of cancer, including PC, among family members. Genetic testing was performed with the identification of pathogenic changes in *ATM*. The subsequent identification of a pathological variant of the *ATM* gene allowed for testing of additional family members. Per the genetic counselor, the *ATM* variant most likely originated through the maternal line, as evidenced by multiple cancer cases on this side of the family. The *ATM* gene responds to DNA damage and produces a serine/threonine kinase that assists in DNA repair, cell-cycle control, and apoptosis [9]. Persons with *ATM* pathogenic variants have increased age-related risk for developing PC at 6.3% and 9.5% by ages 70 and 80 years, respectively, with no apparent differences in penetrance for men and women [10]. Additionally, pedigree analysis identified multiple family members affected by Alzheimer’s disease. There have been suggested associations between the *ATM* gene and Alzheimer’s disease; however, more research is needed [11–13].

## Discussion

### Patient psychological responses to a PC diagnosis

FPC poses significant psychosocial challenges to the patient and his other family members. Persons diagnosed with FPC can have physical symptoms of pain, decreased appetite, and fatigue, accompanied by psychological reactions which include depression and anxiety [14]. Persons with PC have reported more anxiety, distress, and depression than those with other cancer types [15]. The frequent accompaniment of depression and anxiety with pancreatic cancer is not understood [16]. Feelings of depression may precede a diagnosis of PC, with the detection of depression occurring in a majority of persons with PC within six months of diagnosis [17]. It has been suggested that depression and anxiety may stem from biological, metabolic, or functional responses to the illness [16]. Czerw et al. [18] reported greater negative effects of anxiety and helplessness with less fighting spirit among persons with PC than other gastrointestinal cancers. In a large Delphi study of PC patients in treatment, patients prioritized four core psychosocial domains: overall quality of life, relationships with partners and family, satisfaction with providers, and fear of PC recurrence [19]. Information about the heritability of the cancer did not reach consensus as a priority among participants (≤ 60%) in that study.

### The psychosocial impact of FPC on families

The discovery of PC is often devastating for individuals and families who may be blindsided by the diagnosis. PC in a family member can affect the lifestyle and daily habits of at-risk family members across generations. Family members can experience psychosocial distress because they are concerned about their health while simultaneously caring for family members suffering from PC symptoms and declining health. Family members may worry about the genetic risk for themselves and others [20] and have an increased risk perception for PC [1].

### Family members fear of developing PC

Fear can affect the psychological and social aspects of at-risk individuals. Underhill et al. surveyed patients at risk for developing FPC and found that approximately 58% reported they worried about developing PC anywhere from sometimes to all the time. Over half of the participants reported having some degree of worry about developing pancreatic cancer, and over 15% worried often or all the time [1]. This worry can occur throughout the caregiver’s life; however, it was greatest following caring for a family member with PC (within the last five years) or the recent death of family member from PC [1]. Breitkopf et al. found that 81% of people with a family history of FPC reported a degree of concern (anywhere from mild to extreme) in comparison to only 1% of the control group (those without a history of FPC) having concern about developing pancreatic cancer [2].

Researchers also recognized trends in the various times that patients with a history of FPC reported worry. Underhill et al. [1, 7] found that around 57% of participants said that they worried about developing pancreatic cancer when they are going in for screening tests, and around 58% of participants reported worrying when they are thinking about a loved one who has had pancreatic cancer. Other significant times of worry for participants included on their birthday or on the birthday of a loved one who has died from pancreatic cancer. Underhill et al. [7] show comparable results: participants in one-on-one interviews reported having the most “fear and worry” around screening and testing times, “anniversaries of loved ones’ deaths,” growing older, and having other friends surrounding them develop cancer. Underhill et al. [7] also found that when participants of this study thought about their future and dying, it was pancreatic cancer that they feared, not dying of any other cause.

These high percentages of worry show a need for family guidance and counseling when inherited pancreatic cancer is identified. Konings et al. [21] administered psychological screening questionnaires to family members at risk for FPC. The study found that participants with moderately high cancer worry should be thoroughly screened and counseled to improve psychosocial well-being [21]. In summary, people and families experiencing increased worry about developing PC require additional guidance and strategies for psychological support to decrease their concerns and improve their wellbeing.

### Increased risk perception for those at risk for FPC

Those with a family history of FPC not only live with greater fear but also have a much higher perceived lifetime risk of developing pancreatic cancer [1]. In a study by Underhill et al. [1], most participants thought they had a 50% or higher risk of developing pancreatic cancer when the actual risk rarely exceeds 30% and can be as low as 4%. Although those with a family history of pancreatic cancer have an increased risk of developing the disease compared to the general population, they perceive their risk as much higher than the actual one [1]. Breitkopf et al. [2] found similar results when comparing those of the same gender, age, and race. When compared to a control group, 54% of those with a family history of FPC reported they were likely to develop pancreatic cancer at some point in their life. At the same time, only 6% of people without FPC reported a likelihood of having pancreatic cancer in the future [2].

Underhill et al. [7] and Konings et al. [21] found that the family experience of watching a family member ill with pancreatic cancer was highly correlated with inflated risk perceptions. Seeing a family member with the disease had a more significant impact on risk perception than testing positive for having a pathogenic variant associated with pancreatic cancer development. Although people knew they had a higher risk due to genetic mutations, their main reason for worrying was due to the diagnosis of a family member [7, 21]. This inflated risk perception of developing pancreatic cancer affects the lives of those with FPC and calls for greater assistance and supportive care for those diagnosed with FPC [7]. Such discrepancies between perceived and actual risk highlight the importance of advocacy and education programs for at-risk individuals.

### Receptivity to screening and responses to surveillance for FPC

Persons at high risk for FPC may engage in long-term surveillance for early detection of FPC. Individuals more likely to undergo screening for pancreatic cancer had higher rates of cancer worry [2]. Breitkopf et al. [2] found that those with FPC were more receptive to endoscopic ultrasound screenings for pancreatic cancer than those without FPC (41% compared to 16%, respectively). Underhill et al. [7] reported that at-risk individuals were more likely to undergo less invasive screening tests. These individuals always worried about the outcome of their screening tests but still went through screening in hopes of identifying any cancerous changes early [7]. Of note, in a study by Maheu et al. [22], anxiety associated with participation in screening for FPC did not lead to general distress, increased risk perception, and cancer worry. In fact, cancer worry diminished over time which was attributed to counseling and screening activities [22]. O’Neill et al. [4] conducted a study to evaluate the effects of a short and long-term screening program using endoscopic ultrasound and or MRI for pancreatic cancer in a high-risk group (FPC/ BRCA2). Those with personal histories of cancer or positive BRCA2 mutations had increased worry of developing cancer at baseline. However, no negative impact appeared due to screening in the short term. Furthermore, long-term psychological benefits to screening were appreciated in lessening anxiety responses to screening, psychological consequences (emotional, social, and physical domains), and cancer worry [4]. Overbeek et al. [23] examined the burden of surveillance and noted that cancer worries increased during intensified surveillance (lesion identified or had surgery) but decreased with a return to regular surveillance schedules. Overall anxiety and depression measures were unchanged throughout, with quality of life scores in the months following surgery, scores for both physical and mental components were at comparable levels to the public at large [23]. In another study by Overbeek et al. [24], long-term surveillance of at-risk persons found ongoing engagement within the screening program was high.

### Family psychosocial experiences related to FPC

Persons caring for family members with FPC or PC had increased psychosocial burdens related to their experiences of FPC and PC. Breitkopf et al. [2] found that the family experience with pancreatic cancer was the most significant factor related to increased worry. Pancreatic cancer has a lower survival rate than other cancers; therefore, caregivers experience more worry, suffering, guilt, and anticipatory grief as they watch their loved one deal with disease symptoms [20]. Sherman et al. [20] noted a number of salient categories in a qualitative study of caregivers of patients with advanced pancreatic cancer. These included crises related to the diagnosis of cancer, stressors of providing direct care, frustrations of interacting with medical personnel, financial burdens, the constriction of social life and loss of general pleasures in life. Caregivers reported long wait times at appointments, inconsistent medical advice, and sparse information or information overload as being some factors that impacted patient and caregiver experiences [20]. In some instances, study participants gained new insight into their personal strengths and were grateful for time spent with loved ones. However, other caregivers did not cope well and noted psychological withdrawal, stress, depression, and increased drinking and smoking, among other behaviors [20]. A coordinator to help navigate the health care system throughout the patient’s cancer journey could improve patient and family satisfaction with care. Similarly, Kim and Baek [25] reviewed the literature on PC families and found that the diagnosis fueled feelings of fear, stress, depression, anxiety, and helplessness, yet, in some instances, caregivers also reported positive coping mechanisms and enhanced experiences when caring for their loved ones.

Caregivers reported clinically significant levels of anxiety yet claimed better overall quality of life (physical and functional well-being) when compared with those in their care [26]. Age was a factor in this study, with increased anxiety and depression scores among younger (< 60 years) caregivers of persons with PC. A review by Chong et al. [27] revealed select consequences of caregiving included feelings of increased burden in managing a loved one’s symptoms, depression, and anxiety, affecting their quality of life. It also cited an unmet need for better navigation and communication within the health system [27]. Caregivers felt healthcare providers were insensitive, did not provide consistent advice, and provided either too much or too little information [27]. Protocols to guide patient-provider interactions may improve the patient’s and family’s overall satisfaction with care [27].

It is essential to identify individuals at risk for developing FPC and offer psychosocial support to them. Underhill et al. [7] explained that caring for those with FPC includes more than just identifying their risk. Care of these patients also needs to involve psychosocial support and understanding how the knowledge of an increased risk for developing pancreatic cancer can affect one’s life [7].

### Lifestyle changes in at-risk subjects

People at elevated risk for FPC reported increased concern about their health [7]. At-risk individuals wanted to know what lifestyle changes they could make to decrease their risk. Improving their diet, exercising, and reducing tobacco and alcohol consumption were actions at-risk individuals believed could decrease their risk of developing pancreatic cancer. Although people reported wanting to improve their health, others took their increased risk for FPC as a sign to live their life to the fullest and felt it was not worth changing their lifestyle habits [7].

## Clinical implications

Pre-symptomatic and predictive genetic testing are important aspects in the care of individuals and families with a history of FPC. Healthcare providers can play a vital role in discussing these results and counseling patients and their families about risk factors. Additionally, providers can help drive policy that introduces a comprehensive care approach to affected individuals and at-risk family members.

A comprehensive interdisciplinary care team may include a surgical oncologist, medical oncologist, nurse, geneticist or genetic counselor, psychologist, home care or home nursing, social worker, and registered dietician. Involving a care planning team to address needs across family members of varying ages is important to consider. Individuals with the family may require screening, diagnostic procedures, testing, and surveillance. It is important to note this may include young family members who will enter testing and surveillance protocols at some point. Referrals to mental health professionals may be warranted to help patients and families cope with undue stress, anxiety and depression.

It is essential for healthcare providers to identify individuals at risk for developing FPC and offer psychosocial support to them. Psychosocial support is an important component of therapeutic and holistic patient care. Patients and families can feel better supported in their cancer journey through understanding the genetic implications of disease development as well. Underhill et al. [7] explained that caring for those with FPC includes more than just identifying their risk. Future clinical research could focus on the care of these patients and families in improving psychosocial and community supports. Additional research is needed to enhance understanding of the psychosocial effects of an FPC diagnosis on the family.

## Limitations

One family’s experience with FPC was offered as a single case example in this paper. Their experiences may be different from other families living with and caring for persons with the disorder. Research related to the psychosocial responses of patients and families to a diagnosis of FPC/ PC was reviewed but not conducted as a systematic review or an exhaustive search of the literature.

## Conclusions

Pancreatic cancer is the third-leading cause of death from cancer in the United States, with a 95% mortality rate within five years of diagnosis [1]. Early-stage pancreatic cancer is typically asymptomatic. It is often not diagnosed until it is at an advanced stage. To improve pancreatic cancer survivability, better screening for early cancerous changes and an improved understanding of how genetic alterations can predict outcomes are necessary [28]. Screening and genetic testing are recommended for individuals at high risk of developing pancreatic cancer. Performing a comprehensive family history remains an essential tool for identifying individuals at risk for pancreatic cancer [29]. As the case study and review reflects, a known FPC diagnosis can have adverse psychosocial effects on at-risk individuals and families. Providing education, support and screening options to these individuals may help decrease stress and worry and increase coping skills.

## Data Availability

Not applicable.

## Abbreviations

FPC: Familial pancreatic cancer
PC: Pancreatic cancer
COPD: Chronic obstructive pulmonary disease
GERD: Gastroesophageal reflux disease
CT: Computed tomography
ERCP: Endoscopic retrograde cholangiopancreatography
